# The Impact of Supplements on Recovery After Peripheral Nerve Injury: A Review of the Literature

**DOI:** 10.7759/cureus.25135

**Published:** 2022-05-19

**Authors:** Yasmine Abushukur, Rebecca Knackstedt

**Affiliations:** 1 Medicine, Oakland University William Beaumont School of Medicine, Royal Oak, USA; 2 Plastic Surgery, Cleveland Clinic, Cleveland, USA

**Keywords:** peripheral nerve injury, small bowel surgery, postoperative pain, toxic optic neuropathy, neuralgia, vitamins, nerve conduction

## Abstract

Peripheral nerve injury (PNI) can result from trauma, surgical resection, iatrogenic injury, and/or local anesthetic toxicity. Damage to peripheral nerves may result in debilitating weakness, numbness, paresthesia, pain, and/or autonomic instability. As PNI is associated with inflammation and nerve degeneration, means to mitigate this response could result in improved outcomes. Numerous nutrients have been investigated to prevent the negative sequelae of PNI. Alpha-lipoic acid, cytidine diphosphate-choline (CDP Choline), curcumin, melatonin, vitamin B12, and vitamin E have demonstrated notable success in improving recovery following PNI within animal models. While animal studies show ample evidence that various supplements may improve recovery after PNI, similar evidence in human patients is limited. The goal of this review is to analyze supplements that have been used successfully in animal models of PNI to serve as a reference for future studies on human patients. By analyzing supplements that have shown efficacy in animal studies, healthcare providers will have a resource from which to guide decision-making regarding future human studies investigating the role that supplements could play in PNI recovery. Ultimately, establishing a comprehensive understanding of these supplements in human patients following PNI may significantly improve post-surgical outcomes, quality of life, and peripheral nerve regeneration.

## Introduction and background

The peripheral nervous system relays information between the CNS and the remainder of the nervous system outside of the brain and spinal cord [1]. The majority of peripheral nerve injuries (PNI) are secondary to trauma, surgical resection, or toxicity from local anesthetics. Regardless of the cause, severe neuropathic pain is one of the morbidities that can occur due to PNI. Either remove severe neuropathic pain or list the different morbidities that may be associated with PNI [2-5].

Following PNI, a cascade of inflammatory and ischemic molecular events occur in the proximal and distal nerve and can contribute to subsequent neuropathy [6]. PNI results in the formation of free radicals and the release of cytokines. Free radicals increase the permeability of cellular membranes and allow for the intracellular influx of calcium. This influx can lead to the destruction of neurofilaments and microtubules by activating proteolytic pathways [7]. If the free radical damage is allowed to proceed unmitigated, successful nerve regeneration will not occur, resulting in functional or sensory deficit or painful neuropathic pain. If there were means to decrease the inflammatory cascade and quell the free radical production, the extent of injury might not be as great, and recovery could be augmented. Interventions that prevent oxidative stress, neuroinflammation, and cellular injury could be utilized to achieve this. While alternative mechanisms of PNI secondary to surgical trauma may exist, free radical generation and oxidative stress are the most widely understood mechanisms that contribute to PNI at this time.

There is a paucity of human studies investigating nutrients and supplements that may aid peripheral nerve regeneration and recovery. However, animal studies show ample evidence that various supplements may improve recovery after PNI. A review published in 2018 discussed nutrients that may play a role in preserving nerve function and in augmenting recovery after PNI. Nutrients of interest included omega-3 and omega-6 fatty acids, B vitamins, antioxidants, minerals, phenolic compounds, and alpha-lipoic acid [3]. However, numerous supplements which were not previously reviewed in this publication have also demonstrated success in animal models post PNI. The goal of this review is to analyze supplements that have been used successfully in animal models of PNI to serve as a reference for future studies on human patients. By analyzing supplements that have shown efficacy in animal studies, healthcare providers will have a resource from which to guide decision-making regarding future human studies investigating the role that supplements could play in PNI recovery.

## Review

Methods

A PubMed, EMBASE, and Medline search was conducted using the key terms “antioxidant,” “vitamin,” “peripheral nerve injury,” and “sciatic nerve injury.” This search yielded a total of 96 relevant primary studies that pertained to PNI following surgery, from which data were extracted. Methods of PNI included nerve crush, constriction, ligation, and transection. Supplements with four or more citations will be discussed below in alphabetical order.

Results

Alpha-Lipoic Acid

Alpha-lipoic acid (ALA) is a powerful antioxidant and cofactor for many mitochondrial reactions. It is a scavenger of reactive oxygen species and is able to interact with and regenerate other antioxidants, such as vitamin C and E [8]. Six studies, all performed in rat models, addressed the role of ALA after PNI [9-14]. After PNI, ALA was able to increase levels of antioxidants [9], decrease oxidative stress [12], improve recovery of nerve function and conduction velocity [14] and the area of regenerating axon and myelin [13, 14]. Furthermore, given the well-established role of vitamin B12 in attenuating nerve damage in the CNS, some studies compared the efficacy of vitamin B12 and ALA in peripheral nerve regeneration. Compared to vitamin B12, ALA was more effective in improving sciatic functional index values [10] and restructuring the regenerating nerve [11].

Curcumin

Curcumin, the active ingredient of turmeric [15, 16], has been utilized for centuries to treat inflammatory diseases, pain, and trauma [17]. It regulates numerous cell-signaling pathways by modulating transcription factors and kinases for the expression of inflammatory enzymes, cytokines, adhesion molecules, and cell survival proteins [15, 17-20]. In the CNS, curcumin has a wide range of targets and provides numerous neuroprotective effects, including impacting neurotransmitters in the brain, regulating the hypothalamic-pituitary-adrenal axis, improving nerve regeneration, and inhibiting neuronal apoptosis [21-26].

Nine articles addressed the role of curcumin after PNI [27-35], all of which were conducted in a rat model except one mouse study [29].

After PNI, curcumin was able to reduce cell loss [27], improve the action, potential amplitude and conduction velocity [28], improve mechanical sensitivity [29], functional assessments [29, 31, 32, 35], motor and sensitive nerve conduction velocity [29], axonal regeneration [32, 34], myelination [29, 31, 33], and therefore improved the diameter of nerve fibers [31], reduce reactive oxygen species [29, 35], lipid peroxidation [29] and cell death [35], and expedite the reversal of mechanical allodynia [30].

Cytidine 5’-Diphosphocholine (Citicoline/CDP-choline)

Cytidine 5’-diphosphocholine, also known as Citicoline or CDP-choline, is a naturally occurring nucleotide that plays an essential role in phospholipid synthesis [36]. When provided exogenously, CDP-choline divides into choline and cytidine and serve as substrates for phospholipid synthesis [37].

CDP-choline is critical in establishing and maintaining cell membrane structure and protecting neurons during hypoxic and ischemic conditions [38]. Six articles addressed the role of cytidine 5’-diphosphocholine after PNI, all of which were conducted in a rat model [37, 39-43]. CDP-choline administration resulted in decreased scar formation and nerve adherence to surrounding tissue, improved sciatic nerve functional recovery, increased amplitude of the muscle action potential, axonal organization, axonal counts, axonal density, and axonal myelination following PNI [37, 39-43], and reduced neuropathic pain [42]. In addition, CDP-choline decreased levels of MMP-2 and MMP-9 and increased MMP inhibitors TIMP-1 and TIMP-3 [39].

Epigallocatechin-3-Gallate 

Epigallocatechin-3-gallate (EGCG) is the main polyphenolic compound found in Camellia sinensis, also known as green tea. EGCG is a free radical scavenger, oxidative stress inhibitor, modulator of apoptosis, pro-oxidant, and anti-inflammatory agent [44-53]. 

Four articles addressed the role of EGCG after PNI [54-57]. Studies were performed in either rat or rabbit sciatic nerve crush [54,57], rat sciatic nerve transection [55], or rat vagus or hypoglossal crush models [56]. 

EGCG was able to improve the axonal and myelin regeneration, enhance functional recovery [54] and neuronal survival time after transection [55], reduce markers of oxidation [56], alleviate motor and sensory impairment, and improve neuronal regeneration [57].

Melatonin

Melatonin is secreted by the pineal gland at the base of the brain and it plays numerous roles in the human body, including regulating circadian rhythms, sleep physiology, mental status, reproduction, tumor development, and aging [58-60]. In addition, it acts as an antioxidant via a direct influence on toxic radicals and through the induction of enzymes that detoxify free radicals [7]. Thirteen articles addressed the role of melatonin after PNI [7, 61-72], all of which were conducted in a rat model except for two mouse studies [61, 62]. Melatonin improved structural preservation of the myelin sheaths [61], neural regeneration [72], functional outcomes [7, 65, 67], nerve conduction velocity [65], Schwann cell proliferation [64], axonal regeneration [66], increased malondialdehyde [62], decreased nerve peroxidation [63], and reduced oxidative stress [68, 71].

Quercetin

Quercetin, a plant flavonoid found in many fruits, vegetables, and aromatic herbs [73], is a powerful antioxidant, anti-angiogenic, anti-inflammatory, neuroprotective, and anti-apoptotic agent [73, 74]. Three studies addressed the role of quercetin after PNI; two were performed in mouse models [75, 76], and one each was performed in both mouse and rat models [77]. Quercetin enhanced axon remyelination, motor nerve conduction velocity, plantar muscle function [76], and nerve regeneration [77]. It was also found to be superior to gabapentin and morphine in alleviating mechanical and thermal hypersensitivity [75]. 

Vitamin B12

Vitamin B12 is a water-soluble vitamin obtained from dietary meat, eggs, dairy, and other animal-derived products [78]. The deficiency of vitamin B12 can result in neurotoxicity and contribute to the development of subacute combined degeneration, a disorder of the CNS characterized by sensory deficits, motor weakness, paresthesia, and gait ataxia [79]. In addition, within the peripheral nervous system, there is evidence suggesting that B vitamins play a role in peripheral nerve repair following insult [80]. Four studies investigated the role of vitamin B12 after PNI using rat models; two used vitamin B12 in combination with other vitamins [81, 82], and two investigated the role of vitamin B12 alone [80, 83]. Combined B-vitamin administration improved the toe-spreading reflex [81]. Compared to vitamin B1 and B6 alone, vitamin B12 was superior in augmenting peripheral nerve regeneration [80]. A combination of vitamin B12 and vitamin E acetate increased motor nerve conduction velocity and decreased the progression of thermal hyperalgesia following sciatic nerve crush injury [82]. At high doses, methylcobalamin, the active form of vitamin B12, accelerated nerve regeneration, increased myelination, and improved motor and functional recovery of injured nerves [80, 83].

Vitamin E

Vitamin E is an essential lipid-soluble vitamin with potent antioxidant effects. In addition to preventing free-radical reactions, vitamin E can act as a chain-breaking antioxidant that prevents lipid peroxidation [84]. Five articles addressed the role of vitamin E following PNI, all of which were conducted in a rat model [82, 85, 86] except one mouse study [87] and one cat study [88]. Vitamin E administration improved sciatic nerve function, increased the number of functional motor neurons, suppressed cold and mechanical allodynia, and decreased Wallerian degeneration, nerve gliosis, muscle atrophy, blood malondialdehyde levels, and injury-induced 4-hydroxynonenal activity [85,86]. When combined with selenium, it decreased the degeneration of motor nerve terminals and preserved the function of the motor nerve terminals within the soleus muscle [88]. While topical vitamin E alone improved functional sciatic nerve recovery, combined vitamin E and pyrroloquinoline quinone demonstrated significantly stronger benefits to vitamin E alone in nerve conduction velocity, functional motor recovery, and nerve regeneration [87]. 

A summary of supplements with four or more citations is included below in Table 1.

**Table 1:** Summary of reviewed supplements.

Supplement	Outcomes
Alpha-Lipoic Acid	-Increased levels of antioxidants [9]
	-Improved sciatic functional index values [10]
	-Aided in healing and remyelinating damaged nerves [11]
	-Decrease oxidative stress [12]
	-Prevented degeneration of both axons and myelin [13]
	-Improved recovery of nerve function [14]
	-Increased nerve conduction velocity [14]
Curcumin	-Reduced cell loss [27]
	-Improved the action potential amplitude of the sciatic nerve [28]
	-Increased conduction velocity of motor neurons [28]
	-Improved mechanical sensitivity [29]
	-Improved motor and sensitive nerve conduction velocity [29]
	-Improved functional assessments [29, 31, 32, 35]
	-Improved axonal regeneration [32, 34]
	-Increase nerve myelination and therefore the diameter of nerve fibers [29, 31, 33]
	-Reduce reactive oxygen species, lipid peroxidation and cell death [29, 35]
	-Expedited the reversal of mechanical allodynia [30]
Cytidine 5’-diphosphocholine (Citicoline/CDP-choline)	-Decreased levels of MMP-2 and MMP-9 with increased levels of TIMP-1 and TIMP-3 [39]
	-Decreased scar formation [37, 41-43]
	-Decreased nerve adherence to surrounding tissue [37, 40-43]
	-Improved sciatic nerve functional recovery [37, 40-43]
	-Increased amplitude of the muscle action potential [37, 40, 41]
	-Increased axonal organization [37, 43]
	-Increased axons, axonal density, and axonal myelination [37, 39-43]
	-Decreased neuropathic pain [42]
(−)-Epigallocatechin-3-Gallate (EGCG)	-Improved axonal and myelin regeneration [54]
	-Enhanced functional recovery [54]
	-Increased neuronal survival time after transection [55]
	-Reduced markers of oxidation [56]
	-Alleviated motor and sensory impairment [57]
	-Improved neuronal regeneration [57]
Melatonin	-Improved structural preservation of the myelin sheaths [61]
	-Increased malondialdehyde [62]
	-Improved functional outcomes [7, 65, 67]
	-Decreased nerve peroxidation [63]
	-Increased Schwann cell proliferation [64]
	-Increased nerve conduction velocity [65]
	-Increased axonal regeneration [66]
	-Reduced oxidative stress [68]
	-Improved neural regeneration [72]
Quercetin	-Alleviating mechanical and thermal hypersensitivity [75]
	-Enhanced axon remyelination [76]
	-Increased motor nerve conduction velocity [76]
	-Improved plantar muscle function [76]
	-Improved nerve regeneration [77]
Vitamin B12	-Augmented peripheral nerve regeneration [80]
	-Improved toe-spreading reflex when combined with B1 and B6 [81]
	-Increased motor nerve conduction velocity when combined with vitamin E acetate [82]
	-Decreased the progression of thermal hyperalgesia when combined with vitamin E acetate [82]
	-Accelerated nerve regeneration [80, 83]
	-Increased nerve myelination [80, 83]
	-Improved motor and functional recovery of injured nerves [80, 83]
Vitamin E	-Increased functional motor neurons [85]
	-Decreased nerve gliosis [85]
	-Improved sciatic nerve function [85, 86]
	-Reduced muscle atrophy [86]
	-Decreased blood malondialdehyde levels and injury-induced 4-hydroxynonenal activity [86]
	-Decreased cold and mechanical allodynia [86]
	-Decreased Wallerian degeneration [86]
	-Reduced degeneration of motor nerve terminals when combined with selenium [88]
	-Preserved soleus muscle motor nerve terminals when combined with selenium [88]
	-Increased nerve conduction velocity [82, 87]
	-Improved motor functional recovery [87]
	-Improved nerve regeneration [87]

Additional supplements whose roles have been investigated following PNI in animal models include Acetyl-L-carnitine [89], Achyranthes bidentata [90,91], Acorus calamus [92,93], Agmatine [94], Alstonia scholaris [95], Ascorbic Acid [96], Azadirachta Indica [97], Butea monosperma [98], Cannabis sativa [99], Catechin [100], Creatine [101], Crocetin (saffron) [102,103], Crocin [86,103], Diethyldithiocarbamate (DEDC) [104], Elaeagnus angustifolia [105], Frankincense [106], Genistein [107, 108], Ginkgo biloba [109], Glycyrrhizin [110], Green tea [111], Hericium erinaceus [112], Hydroalcoholic extract of red propolis [113], Isoquercitrin [114], Lithium [115], Lumbricus extract [116, 117], Magnesium [118], Ocimum sanctum [119], Pralidoxime [120], Primrose oil [121], Propolis [122], Punica granatum L [123], Pyrroloquinoline quinone [87], Resveratrol [124,125], Radix Hedysari [126], Safranal [86], Salvia officinalis [127], Sesame oil [128], Selenium [88], Soy Phytoestrogens [129], Soybeans [130], Vitamin B1 [80, 81], Vitamin B6 [80, 81], Vitamin D2 [131], and Vitamin D3 [132, 133].

Discussion

PNI can have devastating complications, ranging from functional or sensory deficits to painful neuroma formation. However, numerous supplements have demonstrated success in animal models of PNI to mitigate the inflammatory response and improve regeneration. 

One consideration when translating animal study to human research is to assess the role of a single supplement versus combination therapy. While animal studies have investigated both single supplement and combination therapy, this is not as easily replicated in human research. As numerous supplements have demonstrated success in animal models of PNI, it might stand to reason that the most efficacious approach in humans would be to utilize numerous supplements simultaneously. While this may result in beneficial outcomes, it would remain uncertain which supplement was responsible for the observed results and if the supplements had an unexpected synergistic effect. Despite this uncertainty, the majority of the supplements aforementioned have a low side-effect profile and are generally well-tolerated by humans. Thus, while not clearly delineating the mechanism of action, combination trials in humans may still prove to be the most efficacious approach to optimize results. 

Furthermore, the timing of the intervention was noted to augment healing after PNI. While many of the animal studies reviewed deliver the intervention prior to PNI, this is not always feasible in humans. However, interventions prior to injury are possible in certain scenarios, including amputation with nerve transection and surgeries that have the potential for nerve injury, such as parotidectomy with facial nerve preservation. In these instances, preoperative supplementation might play a synergistic role in a meticulous surgical technique in hastening nerve regeneration/ healing and preventing untoward outcomes. It was beyond the scope of this review to discuss supplementation to augment nerve recovery after nerve grafting and repair, but this is another area that requires investigation.

Also, rodents are metabolically very different from humans. They have a greater amount of metabolically active tissues, such as liver and kidney, and a lesser amount of metabolically inactive tissues, such as bones [134-136]. This could influence the rate of metabolism of supplements. In addition, rodents have different microbiomes than humans as they coevolved with different pathogens [137]. This would impact how rodents respond to various medications and how supplements are metabolized in the gut. Additionally, nerve gaps in rats are very small compared to most human gap lengths, and axotomies in rats can undergo complete recovery, unlike humans [138]. Thus, while animal models can certainly provide valuable information, they need to serve as a nidus for further, well-done human research.

## Conclusions

In summary, numerous antioxidant supplements have demonstrated success in improving recovery after PNI. The mechanism of action is typically mitigation of inflammation and reactive oxygen species production. While these should serve as a nidus for future human trials, there are many important considerations when translating these studies to humans. However, the arena of supplementation to improve PNI in humans is relatively unexplored and requires well-structured prospective studies. 

## References

[1] Lee S, Notterpek L (2013). Dietary restriction supports peripheral nerve health by enhancing endogenous protein quality control mechanisms. Exp Gerontol.

[2] Bouyer-Ferullo S (2013). Preventing perioperative peripheral nerve injuries. AORN J.

[3] Yildiran H, Macit MS, Özata Uyar G (2020). New approach to peripheral nerve injury: nutritional therapy. Nutr Neurosci.

[4] Navarro X, Vivó M, Valero-Cabré A (2007). Neural plasticity after peripheral nerve injury and regeneration. Prog Neurobiol.

[5] Galán-Arriero I, Serrano-Muñoz D, Gómez-Soriano J, Goicoechea C, Taylor J, Velasco A, Ávila-Martín G (2017). The role of Omega-3 and Omega-9 fatty acids for the treatment of neuropathic pain after neurotrauma. Biochim Biophys Acta Biomembr.

[6] Bekar E, Altunkaynak BZ, Balcı K, Aslan G, Ayyıldız M, Kaplan S (2014). Effects of high fat diet induced obesity on peripheral nerve regeneration and levels of GAP 43 and TGF-β in rats. Biotech Histochem.

[7] Atik B, Erkutlu I, Tercan M, Buyukhatipoglu H, Bekerecioglu M, Pence S (2011). The effects of exogenous melatonin on peripheral nerve regeneration and collagen formation in rats. J Surg Res.

[8] Packer L, Tritschler HJ, Wessel K (1997). Neuroprotection by the metabolic antioxidant α-lipoic acid. Free Radic Biol Med.

[9] Kocaoğlu S, Aktaş Ö, Zengi O, Tufan A, Karagöz Güzey F (2018). Effects of alpha lipoic acid on motor function and antioxidant enzyme activity of nerve tissue after sciatic nerve crush injury in rats. Turk Neurosurg.

[10] Horasanli B, Hasturk AE, Arikan M (2017). Comparative evaluation of the electrophysiological, functional and ultrastructural effects of alpha lipoic acid and cyanocobalamin administration in a rat model of sciatic nerve injury. J Back Musculoskelet Rehabil.

[11] Arikan M, Togral G, Hasturk AE (2016). Histomorphometric and ultrastructural evaluation of long-term alpha lipoic acid and vitamin B12 use after experimental sciatic nerve injury in rats. Turk Neurosurg.

[12] Senoglu M, Nacitarhan V, Kurutas EB, Senoglu N, Altun I, Atli Y, Ozbag D (2009). Intraperitoneal alpha-lipoic acid to prevent neural damage after crush injury to the rat sciatic nerve. J Brachial Plex Peripher Nerve Inj.

[13] Demir R, Yayla M, Akpinar E (2014). Protective effects of alpha-lipoic acid on experimental sciatic nerve crush injury in rats: assessed with functional, molecular and electromicroscopic analyses. Int J Neurosci.

[14] Azizi S, Hamidi Alamdari D, Amini K, Raisi A, Azimzadeh M (2015). Alpha-lipoic acid loaded in chitosan conduit enhances sciatic nerve regeneration in rat. Iran J Basic Med Sci.

[15] Goel A, Kunnumakkara AB, Aggarwal BB (2008). Curcumin as "Curecumin": from kitchen to clinic. Biochem Pharmacol.

[16] Nelson KM, Dahlin JL, Bisson J, Graham J, Pauli GF, Walters MA (2017). The essential medicinal chemistry of curcumin. J Med Chem.

[17] Aggarwal BB, Sung B (2009). Pharmacological basis for the role of curcumin in chronic diseases: an age-old spice with modern targets. Trends Pharmacol Sci.

[18] Anand P, Thomas SG, Kunnumakkara AB (2008). Biological activities of curcumin and its analogues (Congeners) made by man and Mother Nature. Biochem Pharmacol.

[19] Menon VP, Sudheer AR (2007). Antioxidant and anti-inflammatory properties of curcumin. Adv Exp Med Biol.

[20] Sandur SK, Pandey MK, Sung B (2007). Curcumin, demethoxycurcumin, bisdemethoxycurcumin, tetrahydrocurcumin and turmerones differentially regulate anti-inflammatory and anti-proliferative responses through a ROS-independent mechanism. Carcinogenesis.

[21] Liu H, Li Z, Qiu D, Gu Q, Lei Q, Mao L (2010). The inhibitory effects of different curcuminoids on β-amyloid protein, β-amyloid precursor protein and β-site amyloid precursor protein cleaving enzyme 1 in swAPP HEK293 cells. Neurosci Lett.

[22] Ma J, Liu J, Yu H, Wang Q, Chen Y, Xiang L (2013). Curcumin promotes nerve regeneration and functional recovery in rat model of nerve crush injury. Neurosci Lett.

[23] Zhang C, Browne A, Child D, Tanzi RE (2010). Curcumin decreases amyloid-β peptide levels by attenuating the maturation of amyloid-β precursor protein. J Biol Chem.

[24] Ahmed T, Gilani AH, Hosseinmardi N, Semnanian S, Enam SA, Fathollahi Y (2011). Curcuminoids rescue long-term potentiation impaired by amyloid peptide in rat hippocampal slices. Synapse.

[25] Jaisin Y, Thampithak A, Meesarapee B (2011). Curcumin I protects the dopaminergic cell line SH-SY5Y from 6-hydroxydopamine-induced neurotoxicity through attenuation of p53-mediated apoptosis. Neurosci Lett.

[26] Stankowska DL, Krishnamoorthy VR, Ellis DZ, Krishnamoorthy RR (2017). Neuroprotective effects of curcumin on endothelin-1 mediated cell death in hippocampal neurons. Nutr Neurosci.

[27] Noorafshan A, Omidi A, Karbalay-Doust S (2011). Curcumin protects the dorsal root ganglion and sciatic nerve after crush in rat. Pathol Res Pract.

[28] Liu GM, Xu K, Li J, Luo YG (2016). Curcumin upregulates S100 expression and improves regeneration of the sciatic nerve following its complete amputation in mice. Neural Regen Res.

[29] Caillaud M, Chantemargue B, Richard L (2018). Local low dose curcumin treatment improves functional recovery and remyelination in a rat model of sciatic nerve crush through inhibition of oxidative stress. Neuropharmacology.

[30] Jeon Y, Kim CE, Jung D (2013). Curcumin could prevent the development of chronic neuropathic pain in rats with peripheral nerve injury. Curr Ther Res Clin Exp.

[31] Moattari M, Moattari F, Kouchesfahani HM, Kaka G, Sadraie SH, Naghdi M, Mansouri K (2018). Curcumin and biodegradable membrane promote nerve regeneration and functional recovery after sciatic nerve transection in adult rats. Ann Plast Surg.

[32] Ma J, Yu H, Liu J, Chen Y, Wang Q, Xiang L (2016). Curcumin promotes nerve regeneration and functional recovery after sciatic nerve crush injury in diabetic rats. Neurosci Lett.

[33] Zhao Z, Li X, Li Q (2017). Curcumin accelerates the repair of sciatic nerve injury in rats through reducing Schwann cells apoptosis and promoting myelinization. Biomed Pharmacother.

[34] Mohammadi R, Mahmoodi H (2013). Improvement of peripheral nerve regeneration following nerve repair by silicone tube filled with curcumin: a preliminary study in the rat model. Int J Surg.

[35] Noorafshan A, Omidi A, Karbalay-Doust S, Aliabadi E, Dehghani F (2011). Effects of curcumin on the dorsal root ganglion structure and functional recovery after sciatic nerve crush in rat. Micron.

[36] Nakazaki E, Mah E, Sanoshy K, Citrolo D, Watanabe F (2021). Citicoline and memory function in healthy older adults: a randomized, double-blind, placebo-controlled clinical trial. J Nutr.

[37] Özay R, Bekar A, Kocaeli H, Karlı N, Filiz G, Ulus IH (2007). Citicoline improves functional recovery, promotes nerve regeneration, and reduces postoperative scarring after peripheral nerve surgery in rats. Surg Neurol.

[38] Secades JJ, Lorenzo JL (2006). Citicoline: pharmacological and clinical review, 2006 update. Methods Find Exp Clin Pharmacol.

[39] Gundogdu EB, Bekar A, Turkyilmaz M, Gumus A, Kafa IM, Cansev M (2016). CDP-choline modulates matrix metalloproteinases in rat sciatic injury. J Surg Res.

[40] Caner B, Kafa MI, Bekar A, Kurt MA, Karli N, Cansev M, Ulus IH (2012). Intraperitoneal administration of CDP-choline or a combination of cytidine plus choline improves nerve regeneration and functional recovery in a rat model of sciatic nerve injury. Neurol Res.

[41] Kaplan T, Kafa IM, Cansev M, Bekar A, Karli N, Taskapilioglu MO, Kanar F (2014). Investigation of the dose-dependency of citicoline effects on nerve regeneration and functional recovery in a rat model of sciatic nerve injury. Turk Neurosurg.

[42] Emril DR, Wibowo S, Meliala L, Susilowati R (2016). Cytidine 5'-diphosphocholine administration prevents peripheral neuropathic pain after sciatic nerve crush injury in rats. J Pain Res.

[43] Aslan E, Kocaeli H, Bekar A, Tolunay S, Ulus IH (2011). CDP-choline and its endogenous metabolites, cytidine and choline, promote the nerve regeneration and improve the functional recovery of injured rat sciatic nerves. Neurol Res.

[44] Sutherland BA, Rahman RM, Appleton I (2006). Mechanisms of action of green tea catechins, with a focus on ischemia-induced neurodegeneration. J Nutr Biochem.

[45] Haque AM, Hashimoto M, Katakura M, Tanabe Y, Hara Y, Shido O (2006). Long-term administration of green tea catechins improves spatial cognition learning ability in rats. J Nutr.

[46] van Praag H, Lucero MJ, Yeo GW (2007). Plant-derived flavanol (-)epicatechin enhances angiogenesis and retention of spatial memory in mice. J Neurosci.

[47] Senggunprai L, Kukongviriyapan V, Prawan A, Kukongviriyapan U (2014). Quercetin and EGCG exhibit chemopreventive effects in cholangiocarcinoma cells via suppression of JAK/STAT signaling pathway. Phytother Res.

[48] Sergent T, Piront N, Meurice J, Toussaint O, Schneider YJ (2010). Anti-inflammatory effects of dietary phenolic compounds in an in vitro model of inflamed human intestinal epithelium. Chem Biol Interact.

[49] Wang T, Zhou H, Xie H, Mu Y, Xu Y, Liu J, Zhang X (2014). Epigallocatechin-3-gallate inhibits TF and TNF-α expression induced by the anti-β2GPI/β2GPI complex in human THP-1 cells. Int J Mol Med.

[50] Higdon JV, Frei B (2003). Tea catechins and polyphenols: health effects, metabolism, and antioxidant functions. Crit Rev Food Sci Nutr.

[51] Mandel S, Weinreb O, Amit T, Youdim MB (2004). Cell signaling pathways in the neuroprotective actions of the green tea polyphenol (-)-epigallocatechin-3-gallate: implications for neurodegenerative diseases. J Neurochem.

[52] Weinreb O, Amit T, Mandel S, Youdim MB (2009). Neuroprotective molecular mechanisms of (-)-epigallocatechin-3-gallate: a reflective outcome of its antioxidant, iron chelating and neuritogenic properties. Genes Nutr.

[53] He M, Zhao L, Wei MJ, Yao WF, Zhao HS, Chen FJ (2009). Neuroprotective effects of (-)-epigallocatechin-3-gallate on aging mice induced by D-galactose. Biol Pharm Bull.

[54] Renno WM, Al-Maghrebi M, Alshammari A, George P (2013). (-)-Epigallocatechin-3-gallate (EGCG) attenuates peripheral nerve degeneration in rat sciatic nerve crush injury. Neurochem Int.

[55] Kian K, Khalatbary AR, Ahmadvand H, Karimpour Malekshah A, Shams Z (2019). Neuroprotective effects of (-)-epigallocatechin-3-gallate (EGCG) against peripheral nerve transection-induced apoptosis. Nutr Neurosci.

[56] Wei IH, Tu HC, Huang CC, Tsai MH, Tseng CY, Shieh JY (2011). (-)-Epigallocatechin gallate attenuates NADPH-d/nNOS expression in motor neurons of rats following peripheral nerve injury. BMC Neurosci.

[57] Renno WM, Benov L, Khan KM (2017). Possible role of antioxidative capacity of (-)-epigallocatechin-3-gallate treatment in morphological and neurobehavioral recovery after sciatic nerve crush injury. J Neurosurg Spine.

[58] Brzezinski A (1997). Melatonin in humans. N Engl J Med.

[59] Cagnacci A (1996). Melatonin in relation to physiology in adult humans. J Pineal Res.

[60] Pierrefiche G, Zerbib R, Laborit H (1993). Anxiolytic activity of melatonin in mice: involvement of benzodiazepine receptors. Res Commun Chem Pathol Pharmacol.

[61] Kaya Y, Sarıkcıoğlu L, Aslan M (2013). Comparison of the beneficial effect of melatonin on recovery after cut and crush sciatic nerve injury: a combined study using functional, electrophysiological, biochemical, and electron microscopic analyses. Childs Nerv Syst.

[62] Kaya Y, Sarikcioglu L, Yildirim FB, Aslan M, Demir N (2013). Does circadian rhythm disruption induced by light-at-night has beneficial effect of melatonin on sciatic nerve injury?. J Chem Neuroanat.

[63] Shokouhi G, Tubbs RS, Shoja MM (2008). Neuroprotective effects of high-dose vs low-dose melatonin after blunt sciatic nerve injury. Childs Nerv Syst.

[64] Chang HM, Liu CH, Hsu WM (2014). Proliferative effects of melatonin on Schwann cells: implication for nerve regeneration following peripheral nerve injury. J Pineal Res.

[65] Rateb EE, Amin SN, El-Tablawy N, Rashed LA, El-Attar S (2017). Effect of melatonin supplemented at the light or dark period on recovery of sciatic nerve injury in rats. EXCLI J.

[66] Kaya Y, Savas K, Sarikcioglu L, Yaras N, Angelov DN (2015). Melatonin leads to axonal regeneration, reduction in oxidative stress, and improved functional recovery following sciatic nerve injury. Curr Neurovasc Res.

[67] Zencirci SG, Bilgin MD, Yaraneri H (2010). Electrophysiological and theoretical analysis of melatonin in peripheral nerve crush injury. J Neurosci Methods.

[68] Chang HM, Ling EA, Lue JH, Wen CY, Shieh JY (2000). Melatonin attenuates neuronal NADPH-d/NOS expression in the hypoglossal nucleus of adult rats following peripheral nerve injury. Brain Res.

[69] Onger ME, Kaplan S, Deniz ÖG (2017). Possible promoting effects of melatonin, leptin and alcar on regeneration of the sciatic nerve. J Chem Neuroanat.

[70] Kaplan S, Pişkin A, Ayyildiz M (2011). The effect of melatonin and platelet gel on sciatic nerve repair: an electrophysiological and stereological study. Microsurgery.

[71] Chang HM, Huang YL, Lan CT, Wu UI, Hu ME, Youn SC (2008). Melatonin preserves superoxide dismutase activity in hypoglossal motoneurons of adult rats following peripheral nerve injury. J Pineal Res.

[72] Moharrami Kasmaie F, Jahromi Z, Gazor R, Zaminy A (2019). Comparison of melatonin and curcumin effect at the light and dark periods on regeneration of sciatic nerve crush injury in rats. EXCLI J.

[73] Waseem M, Parvez S (2016). Neuroprotective activities of curcumin and quercetin with potential relevance to mitochondrial dysfunction induced by oxaliplatin. Protoplasma.

[74] Arikan S, Ersan I, Karaca T, Kara S, Gencer B, Karaboga I, Hasan Ali T (2015). Quercetin protects the retina by reducing apoptosis due to ischemia-reperfusion injury in a rat model. Arq Bras Oftalmol.

[75] Çivi S, Emmez G, Dere ÜA, Börcek AÖ, Emmez H (2016). Effects of quercetin on chronic constriction nerve injury in an experimental rat model. Acta Neurochir (Wien).

[76] Chen MM, Qin J, Chen SJ, Yao LM, Zhang LY, Yin ZQ, Liao H (2017). Quercetin promotes motor and sensory function recovery following sciatic nerve-crush injury in C57BL/6J mice. J Nutr Biochem.

[77] Türedi S, Yuluğ E, Alver A, Bodur A, İnce İ (2018). A morphological and biochemical evaluation of the effects of quercetin on experimental sciatic nerve damage in rats. Exp Ther Med.

[78] Shipton MJ, Thachil J (2015). Vitamin B12 deficiency - A 21st century perspective. Clin Med (Lond).

[79] Qudsiya Z, De Jesus O (2022). Subacute Combined Degeneration of the Spinal Cord. https://pubmed.ncbi.nlm.nih.gov/32644742/.

[80] AL-Saaeed SM, Ali HA, Ali SM, Ali SA (2019). Vitamins B therapy in regeneration of peripheral neuropathy associated with lipid profile. J Phys Conf Ser.

[81] Besalti O, Ergin I, Unlu E, Pekcan Z, Koskan O (2007). The contribution of thiamine, pyridoxine and cyanocobalamine combination on nerve regeneration in rats with experimentally induced sciatic injury. J Anim Vet Adv.

[82] Morani AS, Bodhankar SL (2010). Early co-administration of vitamin E acetate and methylcobalamin improves thermal hyperalgesia and motor nerve conduction velocity following sciatic nerve crush injury in rats. Pharmacol Rep.

[83] Okada K, Tanaka H, Temporin K (2010). Methylcobalamin increases Erk1/2 and Akt activities through the methylation cycle and promotes nerve regeneration in a rat sciatic nerve injury model. Exp Neurol.

[84] Herrera E, Barbas C (2001). Vitamin E: action, metabolism and perspectives. J Physiol Biochem.

[85] Hoshida S, Hatano M, Furukawa M, Ito M (2009). Neuroprotective effects of vitamin E on adult rat motor neurones following facial nerve avulsion. Acta Otolaryngol.

[86] Tamaddonfard E, Farshid AA, Maroufi S (2014). Effects of safranal, a constituent of saffron, and vitamin E on nerve functions and histopathology following crush injury of sciatic nerve in rats. Phytomedicine.

[87] Azizi A, Azizi S, Heshmatian B, Amini K (2014). Improvement of functional recovery of transected peripheral nerve by means of chitosan grafts filled with vitamin E, pyrroloquinoline quinone and their combination. Int J Surg.

[88] Hall ED (1987). Intensive anti-oxidant pretreatment retards motor nerve degeneration. Brain Res.

[89] McKay Hart A, Wiberg M, Terenghi G (2002). Pharmacological enhancement of peripheral nerve regeneration in the rat by systemic acetyl-L-carnitine treatment. Neurosci Lett.

[90] Wang Y, Shen W, Yang L, Zhao H, Gu W, Yuan Y (2013). The protective effects of Achyranthes bidentata polypeptides on rat sciatic nerve crush injury causes modulation of neurotrophic factors. Neurochem Res.

[91] Yuan Y, Shen H, Yao J, Hu N, Ding F, Gu X (2010). The protective effects of Achyranthes bidentata polypeptides in an experimental model of mouse sciatic nerve crush injury. Brain Res Bull.

[92] Muthuraman A, Singh N (2011). Attenuating effect of Acorus calamus extract in chronic constriction injury induced neuropathic pain in rats: an evidence of anti-oxidative, anti-inflammatory, neuroprotective and calcium inhibitory effects. BMC Complement Altern Med.

[93] Muthuraman A, Singh N (2012). Neuroprotective effect of saponin rich extract of Acorus calamus L. in rat model of chronic constriction injury (CCI) of sciatic nerve-induced neuropathic pain. J Ethnopharmacol.

[94] Sezer A, Guclu B, Kazanci B, Cakir M, Coban MK (2014). Neuroprotective effects of agmatine in experimental peripheral nerve injury in rats: a prospective randomized and placebo-controlled trial. Turk Neurosurg.

[95] Singh H, Arora R, Arora S, Singh B (2017). Ameliorative potential of Alstonia scholaris (Linn.) R. Br. against chronic constriction injury-induced neuropathic pain in rats. BMC Complement Altern Med.

[96] Li L, Li Y, Fan Z (2019). Ascorbic acid facilitates neural regeneration after sciatic nerve crush injury. Front Cell Neurosci.

[97] Kandhare AD, Mukherjee AA, Bodhankar SL (2017). Neuroprotective effect of Azadirachta indica standardized extract in partial sciatic nerve injury in rats: evidence from anti-inflammatory, antioxidant and anti-apoptotic studies. EXCLI J.

[98] Thiagarajan VR, Shanmugam P, Krishnan UM, Muthuraman A, Singh N (2012). Ameliorative potential of Butea monosperma on chronic constriction injury of sciatic nerve induced neuropathic pain in rats. An Acad Bras Cienc.

[99] Aziz N, Rasul A, Malik SA, Anwar H (2019). Supplementation of Cannabis sativa L. leaf powder accelerates functional recovery and ameliorates haemoglobin level following an induced injury to sciatic nerve in mouse model. Pak J Pharm Sci.

[100] Yildirim AE, Dalgic A, Divanlioglu D (2015). Biochemical and histopathological effects of catechin on experimental peripheral nerve injuries. Turk Neurosurg.

[101] Helvacioglu F, Kandemir E, Karabacak B (2018). Effect of creatine on rat sciatic nerve injury: a comparative ultrastructural study. Turk Neurosurg.

[102] Wang X, Zhang G, Qiao Y, Feng C, Zhao X (2017). Crocetin attenuates spared nerve injury-induced neuropathic pain in mice. J Pharmacol Sci.

[103] Amin B, Hosseinzadeh H (2012). Evaluation of aqueous and ethanolic extracts of saffron, Crocus sativus L., and its constituents, safranal and crocin in allodynia and hyperalgesia induced by chronic constriction injury model of neuropathic pain in rats. Fitoterapia.

[104] Tariq M, Arshaduddin M, Biary N, Al Deeb S, Al Moutaery K (2000). Diethyldithiocarbamate (DEDC) impairs neuronal recovery following sciatic nerve injury in rats. Restor Neurol Neurosci.

[105] Karimi G, Hosseinzadeh H, Rassoulzadeh M, Razavi BM, Taghiabadi E (2010). Antinociceptive effect of Elaeagnus angustifolia fruits on sciatic nerve ligated mice. Iran J Basic Med Sci.

[106] Jiang X, Ma J, Wei Q (2016). Effect of frankincense extract on nerve recovery in the rat sciatic nerve damage model. Evid Based Complement Alternat Med.

[107] Valsecchi AE, Franchi S, Panerai AE, Sacerdote P, Trovato AE, Colleoni M (2008). Genistein, a natural phytoestrogen from soy, relieves neuropathic pain following chronic constriction sciatic nerve injury in mice: anti-inflammatory and antioxidant activity. J Neurochem.

[108] Ozbek Z, Aydin HE, Kocman AE (2017). Neuroprotective effect of genistein in peripheral nerve injury. Turk Neurosurg.

[109] Al-Adwani DG, Renno WM, Orabi KY (2019). Neurotherapeutic effects of Ginkgo biloba extract and its terpene trilactone, ginkgolide B, on sciatic crush injury model: a new evidence. PLoS One.

[110] Jia YX, Li JR, Mao CY, Yin WT, Jiang RH (2014). Glycyrrhizin improves p75NTR-associated sciatic nerve regeneration in a BALB/c mouse model. Exp Ther Med.

[111] Renno WM, Saleh F, Klepacek I, Al-Khaledi G, Ismael H, Asfar S (2006). Green tea pain modulating effect in sciatic nerve chronic constriction injury rat model. Nutr Neurosci.

[112] Wong KH, Naidu M, David P, Abdulla MA, Abdullah N, Kuppusamy UR, Sabaratnam V (2011). Peripheral nerve regeneration following crush injury to rat peroneal nerve by aqueous extract of medicinal mushroom Hericium erinaceus (Bull.: Fr) Pers. (Aphyllophoromycetideae). Evid Based Complement Alternat Med.

[113] Barbosa RA, Nunes TL, Nunes TL (2016). Hydroalcoholic extract of red propolis promotes functional recovery and axon repair after sciatic nerve injury in rats. Pharm Biol.

[114] Qiu J, Yang X, Wang L (2019). Isoquercitrin promotes peripheral nerve regeneration through inhibiting oxidative stress following sciatic crush injury in mice. Ann Transl Med.

[115] Makoukji J, Belle M, Meffre D (2012). Lithium enhances remyelination of peripheral nerves. Proc Natl Acad Sci USA.

[116] Zhang P, Wang Z, Kou Y (2014). Role of lumbricus extract in the nerve amplification effect during peripheral nerve regeneration. Am J Transl Res.

[117] Wei S, Yin X, Kou Y, Jiang B (2009). Lumbricus extract promotes the regeneration of injured peripheral nerve in rats. J Ethnopharmacol.

[118] Pan HC, Sheu ML, Su HL (2011). Magnesium supplement promotes sciatic nerve regeneration and down-regulates inflammatory response. Magnes Res.

[119] Kaur G, Bali A, Singh N, Jaggi AS (2015). Ameliorative potential of Ocimum sanctum in chronic constriction injury-induced neuropathic pain in rats. An Acad Bras Cienc.

[120] Kaur G, Jaggi AS, Singh N (2010). Ameliorative potential of pralidoxime in tibial and sural nerve transection-induced neuropathic pain in rats. Biol Pharm Bull.

[121] Ramli D, Aziz I, Mohamad M, Abdulahi D, Sanusi J (2017). The changes in rats with sciatic nerve crush injury supplemented with evening primrose oil: behavioural, morphologic, and morphometric analysis. Evid Based Complement Alternat Med.

[122] Yüce S, Cemal Gökçe E, Işkdemir A, Koç ER, Cemil DB, Gökçe A, Sargon MF (2015). An experimental comparison of the effects of propolis, curcumin, and methylprednisolone on crush injuries of the sciatic nerve. Ann Plast Surg.

[123] Jain V, Pareek A, Bhardwaj YR, Singh N (2013). Attenuating effect of standardized fruit extract of Punica granatum L in rat model of tibial and sural nerve transection induced neuropathic pain. BMC Complement Altern Med.

[124] Ding Z, Cao J, Shen Y (2018). Resveratrol promotes nerve regeneration via activation of p300 acetyltransferase-mediated VEGF signaling in a rat model of sciatic nerve crush injury. Front Neurosci.

[125] Bagriyanik HA, Ersoy N, Cetinkaya C (2014). The effects of resveratrol on chronic constriction injury of sciatic nerve in rats. Neurosci Lett.

[126] Wang Z, Zhang P, Kou Y, Yin X, Han N, Jiang B (2013). Hedysari extract improves regeneration after peripheral nerve injury by enhancing the amplification effect. PLoS One.

[127] El Gabbas Z, Bezza K, Laadraoui J (2019). Salvia officinalis, Rosmarinic and Caffeic acids attenuate neuropathic pain and improve function recovery after sciatic nerve chronic constriction in mice. Evid Based Complement Alternat Med.

[128] Hsu CC, Huang HC, Wu PT, Tai TW, Jou IM (2016). Sesame oil improves functional recovery by attenuating nerve oxidative stress in a mouse model of acute peripheral nerve injury: role of Nrf-2. J Nutr Biochem.

[129] Shir Y, Campbell JN, Raja SN, Seltzer Z (2002). The correlation between dietary soy phytoestrogens and neuropathic pain behavior in rats after partial denervation. Anesth Analg.

[130] Pan HC, Cheng FC, Chen CJ (2009). Dietary supplement with fermented soybeans, natto, improved the neurobehavioral deficits after sciatic nerve injury in rats. Neurol Res.

[131] Chabas JF, Stephan D, Marqueste T (2013). Cholecalciferol (vitamin D₃) improves myelination and recovery after nerve injury. PLoS One.

[132] Chabas JF, Alluin O, Rao G (2008). Vitamin D2 potentiates axon regeneration. J Neurotrauma.

[133] Montava M, Garcia S, Mancini J, Jammes Y, Courageot J, Lavieille JP, Feron F (2015). Vitamin D3 potentiates myelination and recovery after facial nerve injury. Eur Arch Otorhinolaryngol.

[134] McNab BK (1997). On the utility of uniformity in the definition of basal rate of metabolism. Physiol Zool.

[135] Agoston DV (2017). How to translate time? The temporal aspect of human and rodent biology. Front Neurol.

[136] Schöch G, Topp H, Held A, Heller-Schöch G, Ballauff A, Manz F, Sander G (1990). Interrelation between whole-body turnover rates of RNA and protein. Eur J Clin Nutr.

[137] Nguyen TL, Vieira-Silva S, Liston A, Raes J (2015). How informative is the mouse for human gut microbiota research?. Dis Model Mech.

[138] Angius D, Wang H, Spinner RJ, Gutierrez-Cotto Y, Yaszemski MJ, Windebank AJ (2012). A systematic review of animal models used to study nerve regeneration in tissue-engineered scaffolds. Biomaterials.

